# S10-4 A comparative evaluation of local governments’ roles in HEPA policy promotion in municipalities from 4 EU countries and Japan

**DOI:** 10.1093/eurpub/ckad133.051

**Published:** 2023-09-11

**Authors:** Petru Sandu, Razvan Mircea Chereches, Antonia Paula Papiu, Peter Gelius, Sven Messing, Anne Vuillemin, Jean-Marie Garbarino, Noriko Takeda, Yukio Oida, Tanja Onatsu, Katariina Tuunanen, Dan Mocan

**Affiliations:** Department of Public Health, University Babes-Bolyai; Department of Public Health, University Babes-Bolyai; Department of Public Health, University Babes-Bolyai; Department of Sport Science and Sport, Friedrich-Alexander-Universität Erlangen-Nürnberg; Department of Sport Science and Sport, Friedrich-Alexander-Universität Erlangen-Nürnberg; Université Côte d'Azur; Université Côte d'Azur; Kogakuin University; Chukyo University; Jamk University of Applied Sciences; Jamk University of Applied Sciences; The BOX - Barbell Club

## Abstract

**Purpose:**

Local governments’ engagement and performance in physical activity promotion is essential to try to reach the nationally and internationally planned population physical activity goals. However, currently, in most countries/settings, local HEPA policy monitoring and evaluation is largely voluntary and often too context specific, using instruments and methods tailored for that specific contexts.

The aim of the Erasmus + Sport co-financed project LoGoPAS was to assess, promote and support local governments' involvement in HEPA (policy) promotion.

**Methods:**

We used complementary qualitative methods (literature review, document analysis, stakeholders’ interviews, stakeholders’ meetings) to analyze and describe the legally binding roles and voluntary based activities of local governments in PA (policy) promotion in selected municipalities from Finland, France, Germany, Japan, and Romania. The data was compiled and presented comparatively between municipalities using an already validated policy monitoring & evaluation tool, the CAPLA- Santé.

**Results:**

The 5 selected municipalities were: Jyväskylä – Finland, Nice – France, Erlangen-Germany, Fujisawa – Japan, Cluj-Napoca – Romania. Following the methods described above and the chapters structure of the CAPLA- Santé we noticed a few similarities (e.g., the existence of a dedicated structure within the municipality, focus of the policy on several population sub-groups, heavy reliance on national or local budgets) and several differences (e.g., the different levels of evidence used in policy development and implementation, differences in the levels and means of monitoring and evaluation) between settings. There are examples of good practice from each setting, e.g. the strength of financing and evaluation in Jyväskylä, the focus on vulnerable populations in Nice, the collaborative approach in Erlangen, the diversity of physical activity promotion programs in Fujisawa and the opportunistic use of external (EU) financing in Cluj-Napoca.

**Conclusion:**

EU and global Comparative analyses of HEPA policy using a validated instrument can offer insight into different approaches, support and stimulate the further engagement of local administrations in physical activity promotion. The efforts to monitor and evaluate physical activity policy should be intensified and potentially/ideally institutionalized.

